# Personal Health Data Spaces for precision prevention and continuity of care: integrating genomic, imaging, and clinical data in primary care

**DOI:** 10.3389/fmed.2026.1903371

**Published:** 2026-08-27

**Authors:** Elisa J. Houwink, Robert R. Freimuth

**Affiliations:** 1 Department of Family Medicine, Mayo Clinic, Rochester, MN, United States; 2 Mayo Clinic College of Medicine and Science, Rochester, MN, United States

**Keywords:** continuity of care, genomics, Personal Health Data Spaces, precision prevention, primary care

## Introduction

Despite substantial advances in disease prevention, current clinical approaches remain largely anchored in short-term risk estimation models that are dominated by age and conventional risk factors. As a result, individuals with substantial inherited susceptibility, particularly younger patients, are frequently underrecognized until disease has already developed. This limitation reflects a broader disconnect between lifetime risk and episodic clinical assessment.

Primary care providers, including general practitioners, family physicians, pediatricians and internal medicine providers, serve as the first point of contact for most patients (depending on the specific health system) and act as gatekeepers to specialized care. With the increasing availability of DNA-based tests and expanding opportunities for genetic screening, these clinicians are increasingly confronted with questions about genetic risk and testing, highlighting the need for better alignment between advances in genomics and the evolving realities of clinical practice. With growing knowledge of the genetic basis of both common chronic diseases (such as diabetes, cancer, and cardiovascular disease) and their monogenic subtypes, including Maturity-Onset Diabetes of the Young (MODY), hereditary breast and ovarian cancer (BRCA1/2), Lynch syndrome, familial hypercholesterolemia (FH), and long QT syndrome (LQTS), as well as recognizing possible rare genetic disorders, polygenic risk scores (PRS) and pharmacogenomic reasons for ineffectiveness or side effects of medications, requires a higher level of genetic literacy in primary care, highlighting the need for better alignment between advances in genomics and the evolving realities of clinical practice.

Studies consistently show that clinicians often lack confidence, practical knowledge, and adequate infrastructure to integrate genetic services into routine care, highlighting the need for better alignment between advances in genomics and everyday clinical practice (1–4). While genomics has been a major driver of personalized medicine, it represents only one component of a broader transformation toward integrating diverse clinical data streams, including imaging, biomarkers, and longitudinal health information, into coherent, actionable insights for patient care.

This article brings together three interrelated developments that are often discussed separately. First, continuity of care remains a defining strength of primary care and an essential prerequisite for effective prevention and chronic disease management. Second, advances in genomics, pharmacogenomics, imaging, and other precision health approaches increasingly enable earlier and more individualized risk assessment, particularly in cardiovascular disease prevention. Third, emerging Personal Health Data Spaces (PHDS) offer a potential infrastructure for integrating these diverse data sources across healthcare settings and over time. We propose that the greatest value of PHDS lies not simply in enabling access to health data, but in supporting continuity of care by connecting multimodal information to longitudinal clinical decision-making. Throughout this article, genomic and cardiovascular prevention examples are used to illustrate how PHDS may help translate fragmented data into actionable insights within routine primary care.

We argue that PHDS should be viewed not primarily as a new source of health data, but as an enabling infrastructure for continuity of care. When implemented within robust governance frameworks, supported by interoperable standards, integrated into multidisciplinary workflows, and designed to promote equity, PHDS may enable precision prevention through the longitudinal integration of genomic, imaging, pharmacogenomic, behavioral, and clinical information. We use cardiovascular and genomic risk assessment as illustrative use cases to examine opportunities, challenges, and implementation requirements.

## Personal Health Data Spaces: actionable layers of personalized data

Personalized medicine, traditionally framed as tailoring (pharmacological) treatment to the individual, is increasingly understood as a broader clinical approach that integrates family history, genomic, and pharmacogenetic information into routine care (5). In primary care practice, this includes recognizing inherited risk patterns, interpreting pharmacogenetic variation in drug response, and incorporating these insights into longitudinal decision-making. Access to an individual's genomic and pharmacogenetic profile provides an additional, actionable layer of personalized data when interpreted within clinical knowledge frameworks and guideline-based decision-making. Beyond optimizing therapy, such data may also enable earlier identification of disease risk and support presymptomatic and preventive strategies, particularly for hereditary conditions such as cancer syndromes and familial cardiovascular disease, as well as common chronic diseases including diabetes (6). Importantly, this approach is also relevant for population-based genetic conditions, such as hemoglobinopathies, where early identification in primary care can facilitate targeted screening (preconceptional) counseling, cascade screening and timely intervention. Together, these developments illustrate how personalized medicine extends beyond isolated tests to a longitudinal, integrated model of care that aligns closely with the principles of continuity and coordination inherent to primary care.

Genomic medicine offers an opportunity to address this gap. Both monogenic conditions, such as FH, and polygenic susceptibility captured through PRS demonstrate that a significant proportion of cardiovascular risk is present from birth and accumulates over time (7, 8). However, while genetic information can identify predisposition, it does not consistently translate into clinical action (6, 9). At the same time, advances in imaging, particularly coronary artery calcium (CAC) scoring and coronary CT angiography (CCTA), allow direct assessment of subclinical disease, providing a bridge between latent risk and phenotypic expression. Yet these data streams remain fragmented, often interpreted in isolation rather than as part of a longitudinal, integrated risk model (10). These challenges extend across chronic conditions. The core issue is not the absence of data, but the lack of integration. Genomic results, imaging findings, laboratory data, and clinical information are often generated in parallel but remain fragmented and difficult to interpret within routine workflows.

Personal health data spaces (PHDS) provide a conceptual framework to address this challenge by integrating multimodal data into a continuous, patient-centered environment. For the purposes of this article, a PHDS refers to a patient-centered, interoperable environment that may enable aggregation, governance, sharing, and longitudinal use of multimodal health data originating from multiple sources. Unlike electronic health records, which are typically organization-centric, PHDS are intended to be person-centric and span organizational boundaries. Similarly, PHDS extend beyond patient portals and personal health records by enabling structured integration of genomic, imaging, clinical, and behavioral information, together with governance mechanisms that support data sharing and reuse. PHDS are also distinct from the European Health Data Space (EHDS), which provides a regulatory and infrastructural framework for data exchange at a societal level; PHDS describe the patient-level environment in which such data can be integrated and used for person- and patient-centric care (11).

## What is a Personal Health Data Space?

For the purposes of this article, we regard a *Personal Health Data Space* (PHDS) as a patient-centered, interoperable environment that enables the longitudinal aggregation, governance, sharing, and use of health-related data originating from multiple sources. PHDS should be viewed as a conceptual model rather than a single technology platform. Its primary purpose is to support continuity of care by making relevant information available across healthcare settings, time points, and clinical disciplines while maintaining appropriate governance, security, and patient involvement.

A PHDS may be implemented through different technical architectures. In a *repository model*, health data are copied or deposited into a centralized personal data environment. In a *federated model*, data remain within the source systems and are accessed when needed through interoperable connections. A *pointer- or index-based model* provides metadata and access pathways that link distributed data sources without necessarily storing the underlying data within the PHDS itself. *Hybrid approaches* combining these models are also possible.

Regardless of architecture, several core components are required. These include a patient-facing access and control layer; identity management mechanisms to ensure accurate individual matching; consent management capabilities governing authorization and withdrawal of access; an interoperability layer supporting syntactic, semantic, and organizational interoperability; clinical decision-support functions that transform data into actionable information; and an audit and governance layer that enables transparency, accountability, provenance tracking, and security oversight.

Potential data inputs to a PHDS could include electronic health records (EHRs), laboratory results, imaging reports and images, genomic and pharmacogenomic data, pharmacy records, family history information, and patient-generated data from questionnaires, wearables, or other digital health tools. These data are integrated to create a longitudinal view of health and disease that extends beyond the boundaries of individual healthcare organizations.

The outputs of a PHDS may include clinician-facing decision support, patient-facing risk and treatment information, and tools that facilitate continuity of care across providers and care transitions. Importantly, the value of PHDS lies not merely in data aggregation but in enabling the appropriate interpretation and use of information at the point of care.

As with any complex health data infrastructure, PHDS may also introduce potential failure modes. These include incomplete or inaccurate data, identity-matching errors, poor interoperability between systems, algorithmic bias in analytic tools, cybersecurity vulnerabilities, and increased clinician burden arising from information overload. Recognition of these limitations underscores the need for robust governance, ongoing evaluation, and careful implementation.

Consider a 38-year-old male with a strong family history of premature coronary disease. Within a PHDS, family history, lipid measurements, prior clinical assessments, genomic information where clinically indicated, and relevant imaging findings, if obtained through guideline-concordant care, could be integrated longitudinally. The combined information could support individualized risk assessment, guide preventive therapy decisions, identify at-risk relatives for cascade screening, and facilitate ongoing shared decision-making within primary care. Realizing these benefits will require effective implementation of PHDS within robust clinical and governance frameworks that clearly define rights of access, data stewardship responsibilities, consent management processes, and accountability structures. Dynamic consent models may provide one approach for enabling individuals to control how genomic and other sensitive information are shared and reused over time. Transparent oversight and auditability will be essential to maintaining public trust (12).

Beyond data integration alone, an additional challenge lies in how data are translated into meaningful predictions. Many clinical data points represent proxies of underlying biological processes. Advances in computational modeling and systems biology may enable more precise prediction of disease trajectories by linking genomic variation to downstream biological pathways. Future analytical approaches, including digital-twin concepts, may eventually build on PHDS infrastructures, although such applications remain largely investigational.

Within such systems, diverse data sources are combined and contextualized over time, enabling clinicians to move beyond isolated risk indicators toward longitudinal, actionable insight. A practical barrier is the fragmentation of data across healthcare systems, where relevant information is often stored in separate, non-interoperable platforms. Practical implementation and integration of PHDS thus requires adoption of interoperability and data-exchange standards. Examples include HL7 FHIR for structured exchange of clinical information, FAIR data principles to improve data discoverability and reuse, and semantic interoperability frameworks that enable consistent interpretation of information across systems. Such standards provide the technical foundation necessary for integrating genomic, imaging, and clinical data into computable decision-support environments enabling implementation in clinical care practice.

Polygenic risk scores (PRS) extend this concept by aggregating the effects of many common genetic variants. Individuals in the highest PRS categories, for example, have been shown to carry significantly increased CAD risk, often comparable in magnitude to monogenic disorders in aggregate effect, particularly when combined with environmental exposures (13, 14). A key advantage of PRS is their ability to identify risk early in life, before conventional risk factors emerge (15).

## Limitations of polygenic risk scores

Despite their potential, several important limitations currently constrain the clinical implementation of PRS. First, many PRS have been developed and validated predominantly in populations of European ancestry, resulting in reduced predictive performance and poorer calibration when applied to individuals from other ancestral backgrounds highlighting concerns regarding equity and generalizability (15–17).

Second, challenges remain regarding calibration and interpretation. While PRS may identify relative differences in inherited susceptibility, translating these estimates into meaningful absolute risk assessments that can guide individual clinical decisions remains complex. Risk estimates may vary across populations, healthcare settings, and underlying disease incidence rates.

Third, uncertainty persists regarding appropriate clinical action thresholds. Although individuals in the highest PRS categories often demonstrate increased disease risk, consensus is still evolving regarding when and how PRS results should alter screening strategies, preventive interventions, or pharmacotherapy. Clinical utility therefore depends not only on predictive performance but also on the existence of evidence-based management pathways.

Fourth, prospective implementation evidence remains limited. While observational studies demonstrate associations between PRS and disease risk, fewer studies have evaluated whether incorporating PRS into routine clinical care improves patient outcomes, changes clinician behavior, or enhances decision-making in real-world healthcare settings.

Finally, the incremental value of PRS beyond established risk assessment approaches remains an area of active investigation. Traditional risk factors, family history, socioeconomic conditions, lifestyle factors, and environmental exposures continue to play major roles in disease development. Consequently, PRS should be viewed as a complementary component of multimodal risk assessment rather than a replacement for established clinical evaluation. Within a PHDS framework, the greatest value of PRS may lie in their integration with other sources of information, allowing inherited risk to be interpreted alongside clinical, behavioral, imaging, and environmental factors (5, 15, 18).

## Genetic risk including PRS as a lifelong determinant of disease

We already practice genetic medicine in routine care, but we do so imprecisely and advancing PHDS could enhance care continuity if integrated effectively. Genetic risk assessment in clinical practice has traditionally relied on family history, which remains a fundamental and accessible tool in primary care. Family history captures shared genetic, environmental, and behavioral factors and often represents the first indication of inherited disease risk. However, it is inherently imprecise, subject to incomplete knowledge, recall bias, and variability in interpretation (19, 20). In contrast, PRS provide a quantitative measure of inherited susceptibility based on the cumulative effect of common genetic variants (21). Rather than replacing family history, PRS should be understood as a complementary tool that refines and objectifies inherited risk. This is particularly relevant in situations where family history is unavailable or incomplete, such as in small families, adoption, estrangement, or conditions associated with stigma, where genetic risk may otherwise go unrecognized. Evidence suggests that PRS and family history capture overlapping but also distinct components of risk, with PRS offering particular value in identifying individuals at increased risk in the absence of a clear family history, and family history providing important context that extends beyond genomic data alone. Integrating both within a longitudinal care framework allows for a more nuanced understanding of risk, particularly in primary care where decisions must account for uncertainty, patient context, and evolving information over time.

A consistent theme across genomic medicine is that disease risk begins long before clinical presentation. In coronary artery disease (CAD), heritability estimates of approximately 40%−60% underscore the importance of inherited factors in shaping lifetime risk. FH represents the most clearly actionable example. Despite its relatively high prevalence (approximately one in 250–300 individuals), FH remains markedly underdiagnosed worldwide (19). Importantly, individuals with pathogenic variants have substantially elevated cardiovascular risk compared to those with similar cholesterol levels but without a genetic cause, demonstrating that genetic information adds clinically meaningful stratification beyond traditional biomarkers (20).

Both FH and PRS illustrate that inherited risk exists along a spectrum and represent distinct forms of inherited risk. FH represents a highly penetrant monogenic condition with established management pathways, whereas PRS estimate susceptibility across a continuum of risk. Importantly, neither form of inherited risk is fully deterministic, and clinical decisions should be informed by the broader clinical context, including family history, conventional risk factors, lifestyle, and where appropriate, imaging findings. Both may contribute to risk stratification, although their evidentiary basis, interpretation, and clinical applications differ substantially.

The presence of a genetic predisposition such as FH and PRS, does not indicate when disease will manifest, or whether it will manifest at all. This creates two challenges: understanding how genetic risk translates into disease development over time and determining how that information should inform clinical decisions. The presence of genetic risk does not necessarily require a change in management; rather, it should support shared decision making that are aligned with an individual's complete genetic and clinical profile (11, 18). Real-world studies and pragmatic trials illustrate that timing, integration into clinical workflows, clinician engagement and patient understanding are critical determinants of impact (2, 11, 12). When genetic information is delivered after treatment decisions have been made, or without clear pathways for action, it may confirm existing decisions or add confusion rather than prompt change. These findings highlight that the clinical utility of genomic data depends not only on its validity, but on *how* and *when* it is integrated into care.

## Multimodal data: linking genetic risk to disease expression

Imaging provides one example of how multimodal data can complement genetic risk assessment by identifying subclinical disease. Genetic and imaging data reflect complementary dimensions of risk: inherited predisposition and phenotypic expression. Imaging modalities such as coronary artery calcium (CAC) scoring integrate lifetime exposure to risk factors and improve prediction of cardiovascular disease (22). Imaging may assist risk reclassification and inform therapeutic decision-making in selected individuals with elevated inherited risk where disease manifestation is uncertain (23).

Early efforts within clinical innovation environments, including those embedded in academic health systems, demonstrate the feasibility of integrating genomic, pharmacogenetic, and clinical data into longitudinal care pathways. Emerging innovation environments further illustrate how such integration may be operationalized in practice. This could make PHDS unique beyond EHRs and EHDS as the distinguishing feature of PHDS would not merely be the availability of health data, but the ability to organize diverse data streams around an individual across time, institutions, and care settings in a manner that supports both patient participation and actionable clinical decision-making.

## From data silos to Personal Health Data Spaces

**Figure 1:**
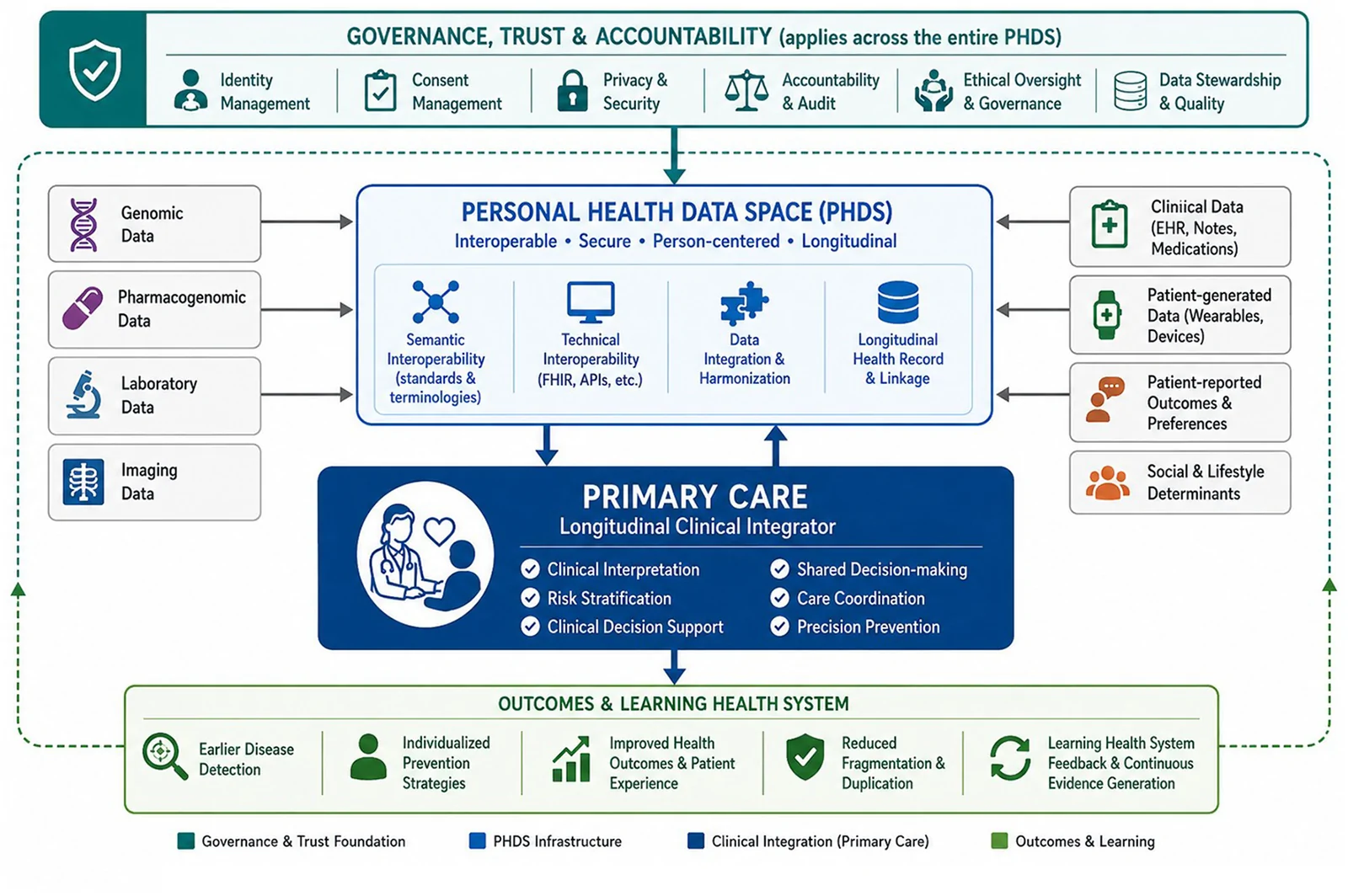
Conceptual model of Personal Health Data Spaces (PHDS) supporting continuity of care and precision prevention. Personal Health Data Spaces (PHDS) integrate multimodal data from diverse sources, including genomic, pharmacogenomic, imaging, laboratory, clinical, patient-generated, and other health-related information. A governance and trust layer—including identity management, consent management, privacy, security, accountability, and audit mechanisms—supports responsible data stewardship across healthcare settings. Interoperability services enable semantic and technical integration of heterogeneous data into longitudinal, person-centered health records. Within this framework, primary care functions as the longitudinal clinical integrator, coordinating multidisciplinary care and translating integrated information into clinical interpretation, shared decision-making, and clinical decision support. These processes are intended to facilitate earlier disease detection, individualized prevention strategies, improved health outcomes, and continuous learning through feedback into a learning health system. These anticipated outcomes represent the proposed potential of PHDS and require prospective evaluation (Figure created with assistance from ChatGPT (OpenAI) and verified by the author). PHDS, Personal Health Data Spaces; EHR, electronic health records; FHIR, fast healthcare interoperability resources; APIs, application programming interfaces.

Within primary care, collaborative models are particularly relevant, as they provide a mechanism to support clinicians in managing genomic complexity without requiring full specialization. By embedding expertise within team-based structures, they may enable more distributed, yet coordinated, approaches to genomic care. In this context, collaborative care models can be understood as part of the infrastructure required to support the mainstreaming of genomics into routine practice (Figure 1).

Interdisciplinary initiatives within clinical innovation laboratories increasingly focus on connecting genomic data, electronic health records, imaging outputs, and clinical decision support into unified, longitudinal platforms. Early examples of such approaches in primary care include the use of personal genomic data infrastructures, such as the Personal Genetic Locker, which demonstrate how pharmacogenomic, clinical, and contextual data can be integrated into decision-support environments while addressing ethical and governance challenges (11, 12, 24).

The development of PHDS also aligns with broader regulatory initiatives aimed at improving health data accessibility and interoperability. In Europe, the European Health Data Space (EHDS), established through Regulation (EU) 2025/327, provides a regulatory and infrastructural framework intended to facilitate the secure exchange and reuse of electronic health data across Member States for both primary and secondary purposes of use. However, EHDS and PHDS represent complementary rather than identical concepts.

EHDS primarily operates at the system and policy level, establishing common rules, governance mechanisms, interoperability requirements, and infrastructures that support the availability and exchange of health data across organizations and jurisdictions. In contrast, PHDS operate at the individual patient level, focusing on the longitudinal integration, governance, and meaningful use of multimodal health information throughout a person's healthcare journey. In practical terms, EHDS may enable health data to become accessible across healthcare systems, whereas PHDS provide a patient-centered environment in which those data can be organized, interpreted, and applied to support continuity of care, shared decision-making, and precision prevention. Thus, *EHDS may be viewed as an enabling ecosystem for data availability, while PHDS represent a complementary clinical and patient-centered framework for longitudinal use of those data*. This distinction is particularly relevant for precision prevention. Access to data alone does not ensure improvements in care. Clinical value ultimately depends on the ability to integrate genomic, imaging, laboratory, pharmacogenomic, and other health information into workflows that support timely interpretation and action by patients and healthcare professionals (7, 8).

Emerging patient-centered data infrastructures are supported by empirical studies showing that both patients and healthcare professionals value control, trust, and interoperability in personal health data spaces, while recognizing implementation challenges (11, 12). This transition reflects two parallel shifts: from fragmented data toward integration, and from population-based estimation toward truly personalized care.

The coexistence of genomic and imaging data highlights a systemic limitation in healthcare: these data streams are generated but not meaningfully integrated. Genetic testing results, imaging findings, and clinical data often remain siloed, reducing their clinical impact.

This fragmentation extends beyond cardiovascular care. Pharmacogenomics, for example, provides actionable insights into drug response, yet its clinical implementation remains limited by lack of integration into clinical workflows and decision support systems. Similar challenges are seen in cancer genetics and metabolic disease management. Importantly, implementation studies show that (pharmaco)genomic data have the greatest clinical impact when available preemptively and embedded within clinical decision support, rather than delivered reactively after prescribing decisions have been made. This creates a translational gap between identifying risk and determining appropriate clinical action. This gap is well-recognized in implementation science, where the impact of complex interventions is often limited not by efficacy, but by challenges in integration into routine workflows and clinical behavior (25, 26).

These approaches reflect the foundational principles of PHDS, where heterogeneous data are structured and made accessible for clinical decision-making and longitudinal care (11).

## Primary care as the longitudinal integrator

In this evolving landscape, primary care shifts from a gatekeeper role to a longitudinal integrator of multimodal health data across conditions and time and thus plays a central role in realizing integrated, data-driven care. As the first point of contact and coordinator of ongoing care, primary care clinicians are uniquely positioned to connect diverse data sources and translate them into meaningful clinical decisions. However, implementation challenges remain prominent, including limited genomic literacy, lack of infrastructure, and fragmented care pathways. In addition, primary care clinicians often remain uncertain about their role in delivering genomic medicine (27). Experiences from pharmacogenomics demonstrate that successful integration requires not only evidence, but also education, workflow integration, and AI/ML and decision support embedded in routine care (3). This integration also depends on the availability of structured, computable data and standardized representations of clinical and genomic information, enabling effective use within decision support systems.

## Discussion

The limited impact of precision medicine in primary care reflects a consistent pattern: implementation challenges often outweigh technological advances (25, 26). Genomic and other complex data are frequently introduced too late in the clinical pathway, insufficiently integrated into decision support systems, or poorly aligned with existing workflows. As a result, even clinically relevant information may not lead to changes in care (12).

This highlights a central principle: the value of data lies not only in its availability, but also in its ability to inform timely and actionable decisions. PHDS offer a potential solution by aligning multimodal data with clinical processes and enabling longitudinal interpretation at the point of care. EHDS makes data available, while PHDS aims to make data clinically useful across time and settings, thereby supporting continuity of care.

## Implementation considerations: a NASSS perspective

Implementation science suggests that the success of health innovations depends not only on technical performance but also on their ability to become embedded within routine practice. The Non-adoption, Abandonment, Scale-up, Spread, and Sustainability (NASSS) framework provides a useful lens through which to examine the opportunities and challenges associated with PHDS implementation.

From a *condition* perspective, PHDS are primarily intended to support the management and prevention of complex chronic diseases, such as cardiovascular disease, diabetes, cancer, and rare genetic disorders, where relevant information is distributed across multiple providers, settings, and time points. From a *technology* perspective, achieving interoperability across heterogeneous systems remains a major challenge, requiring attention to *syntactic, semantic*, and *organizational interoperability*. These interoperability types can be distinguished as follows: *Syntactic interoperability* refers to the technical exchange of data between systems, S*emantic interoperability* ensures that exchanged information retains the same meaning across systems, and *organizational interoperability* encompasses the governance, policies, and workflows that enable effective data sharing and use.

The *value proposition* of PHDS depends on whether integration of multimodal data translates into meaningful clinical utility, improved decision-making, better patient experiences, and ultimately improved health outcomes. For *adopters*, both healthcare professionals and patients must perceive sufficient value to justify changes in established workflows and behaviors. Clinicians may require new competencies in genomic medicine and data interpretation, while patients need understandable interfaces, transparent governance, and confidence in the security and appropriate use of their health information.

At the *organizational level*, implementation will likely require workflow redesign, integration with existing electronic health record systems, multidisciplinary collaboration, and investment in workforce training and clinical decision-support tools. At the broader *system level*, regulatory frameworks, reimbursement mechanisms, data governance requirements, and interoperability standards will influence adoption and scalability. Finally, *the adaptation and sustainability domain* highlights the importance of ongoing evaluation, continuous refinement, and long-term maintenance. PHDS must therefore be capable of evolving alongside advances in genomics, imaging, artificial intelligence, and healthcare delivery while remaining acceptable and useful to patients and healthcare professionals.

Viewed through the NASSS framework, PHDS should thus be considered not merely as a technical intervention but as a complex sociotechnical innovation whose success depends on coordinated action across clinical, organizational, technological, and policy domains. Future evaluations of PHDS should therefore examine not only technical performance, but also continuity of care, patient experience, clinician workload, equity, safety, adoption, and sustainability.

Cardiovascular disease further illustrates this opportunity. Genetic risk identifies early susceptibility; imaging captures phenotypic expression, and primary care provides the longitudinal context for interpretation. When integrated, these elements support a shift from reactive management toward proactive prevention. More broadly, this paradigm extends beyond cardiovascular disease to other chronic conditions, including cancer, pharmacogenomics-informed prescribing, diabetes, and rare diseases, where fragmented data and disconnected care pathways similarly limit the translation of information into clinical action. PHDS provide a unifying framework through which genomic, imaging, laboratory, medication, and patient-generated data can be integrated across settings and over time. By supporting continuity of care and longitudinal interpretation of multimodal information, PHDS may help transform isolated data points into actionable insights that enable more person-centered and proactive approaches to prevention and disease management.

## Recommendations for advancing PHDS in precision prevention

To realize the potential of Personal Health Data Spaces (PHDS) for precision prevention, implementation efforts should focus on several key priorities.

First, continuity of care should be recognized as a primary objective of PHDS development. Data infrastructures should facilitate longitudinal care across providers, organizations, and care transitions, rather than creating additional information silos.

Second, PHDS should be built on interoperable standards and governance frameworks. Technical interoperability standards, including HL7 FHIR and semantic terminologies, should be combined with clear policies for data stewardship, consent management, cybersecurity, accountability, auditing, and data provenance.

Third, PHDS should be embedded within multidisciplinary models of care. Effective implementation will require collaboration among primary care clinicians, genetic specialists, pharmacists, radiologists, data stewards, and technology providers, supported by education, clinical decision support, and workflow redesign.

Fourth, equity should be treated as a core design principle rather than an afterthought. PHDS initiatives should actively address digital exclusion, variability in access to genomic services, health literacy, and the risk of widening existing health disparities.

Finally, future research should move beyond technical feasibility and evaluate whether PHDS improve outcomes that matter to patients, clinicians, and healthcare systems. Priority evaluation domains include continuity of care, patient experience, clinical outcomes, safety, clinician workload, equity, scalability, and long-term sustainability.

## Conclusion

Personal Health Data Spaces should be viewed not primarily as data repositories, but as infrastructures that support continuity of care across time, settings, and healthcare professionals. By supporting the longitudinal integration of genomic, imaging, pharmacogenomic, clinical, and patient-generated data, PHDS have the potential to bridge the gap between fragmented information and actionable clinical insight. However, realizing this potential will require interoperable technologies, robust governance, multidisciplinary collaboration, attention to equity, and rigorous evaluation. Under these conditions, PHDS may help support precision prevention by enabling more coordinated, person-centered, and proactive approaches to health and disease management.
